# Lipid-Polymer Hybrid Nanoparticles Enhance the Potency of Ampicillin against *Enterococcus faecalis* in a Protozoa Infection Model

**DOI:** 10.1021/acsinfecdis.0c00774

**Published:** 2021-04-19

**Authors:** Chuan Hao Tan, Lai Jiang, Wenrui Li, Siew Herng Chan, Jong-Suep Baek, Noele Kai Jing Ng, Talgat Sailov, Sharad Kharel, Kelvin Kian Long Chong, Say Chye Joachim Loo

**Affiliations:** † Singapore Centre for Environmental Life Sciences Engineering, Nanyang Technological University, 60 Nanyang Drive, Singapore 637551; ‡ School of Materials Science & Engineering, Nanyang Technological University, 50 Nanyang Ave, Singapore, 639798; § NTU Institute for Health Technologies, Interdisciplinary Graduate Program, Nanyang Technological University, 61 Nanyang Drive, Singapore 637335; ∥ Harvard T.H. Chan School of Public Health, 677 Huntington Ave, Boston, Massachusetts 02115, United States

**Keywords:** *Enterococcus faecalis*, protozoa, biofilm, intracellular infection, lipid-polymer hybrid nanoparticle, antibiotics

## Abstract

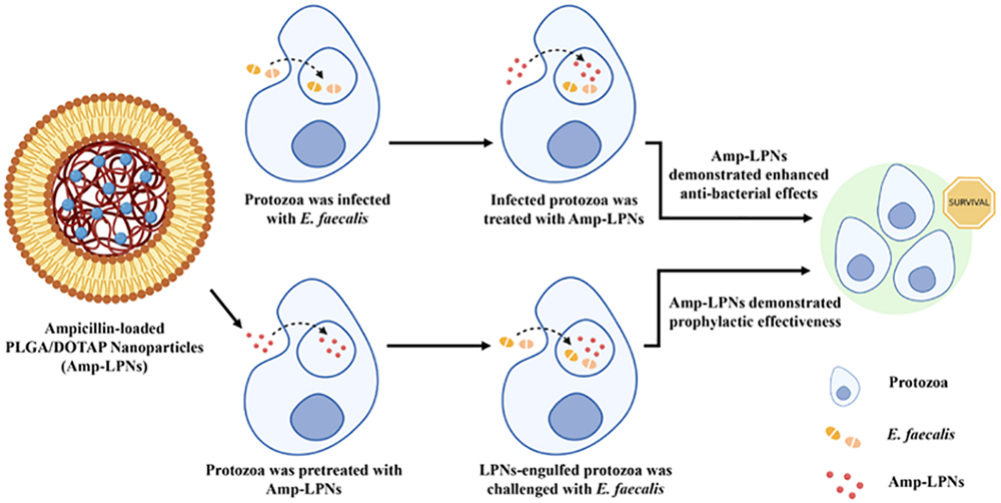

*Enterococcus faecalis* (*E. faecalis*) biofilms are implicated in endocarditis, urinary tract infections, and biliary tract infections. Coupled with *E. faecalis* internalization into host cells, this opportunistic pathogen poses great challenges to conventional antibiotic therapy. The inability of ampicillin (Amp) to eradicate bacteria hidden in biofilms and intracellular niches greatly reduces its efficacy against complicated *E. faecalis* infections. To enhance the potency of Amp against different forms of *E. faecalis* infections, Amp was loaded into Lipid-Polymer hybrid Nanoparticles (LPNs), a highly efficient nano delivery platform consisting of a unique combination of DOTAP lipid shell and PLGA polymeric core. The antibacterial activity of these nanoparticles (Amp-LPNs) was investigated in a protozoa infection model, achieving a much higher multiplicity of infection (MOI) compared with studies using animal phagocytes. A significant reduction of total *E. faecalis* was observed in all groups receiving 250 μg/mL Amp-LPNs compared with groups receiving the same concentration of free Amp during three different interventions, simulating acute and chronic infections and prophylaxis. In early intervention, no viable *E. faecalis* was observed after 3 h LPNs treatment whereas free Amp did not clear *E. faecalis* after 24 h treatment. Amp-LPNs also greatly enhanced the antibacterial activity of Amp at late intervention and boosted the survival rate of protozoa approaching 400%, where no viable protozoa were identified in the free Amp groups at the 40 h postinfection treatment time point. Prophylactic effectiveness with Amp-LPNs at a concentration of 250 μg/mL was exhibited in both bacteria elimination and protozoa survival toward subsequent infections. Using protozoa as a surrogate model for animal phagocytes to study high MOI infections, this study suggests that LPN-formulated antibiotics hold the potential to significantly improve the therapeutic outcome in highly complicated bacterial infections.

Enterococcus faecalis is a diplococcal-shaped Gram-positive bacterium that is part of the normal flora of human gut. This bacterium is a nonspore forming facultative anaerobe tolerant to extreme conditions such as high salinity, pH, temperatures, and bile salts.^1,2^*E. faecalis* is an opportunistic pathogen that causes a wide range of nosocomial infections. Being the second most common cause of infective endocarditis, *E. faecalis* infects the heart valves and forms biofilms (i.e., “vegetations”) mostly in people with cardiovascular conditions.^3−5^ It is also seen in people with urinary catheters, colonizing and forming biofilms on the catheter surface.^6−8^ Conventionally, *E. faecalis* infections are treated with ampicillin (Amp) despite the fact that all enterococci have decreased susceptibility to penicillin and Amp intrinsically due to the production of low-affinity penicillin-binding proteins.^9−11^ In cases where the optimal treatment conditions for *E. faecalis* infections cannot be met, such as in biofilms and intracellular niches, antibiotic therapy fails and leads to intractable chronic infections.

The presence of biofilms is responsible for the persistence and recurrence of various *E. faecalis* infections. A biofilm is a collection of microorganisms residing in a matrix of secreted extracellular polymeric substances (EPS) consisting of polysaccharides, proteins, enzymes, and DNA that acts as a physical barrier.^12,13^ It not only retards the penetration of Amp but also shields the bacteria from the host immune attacks. Besides its capability of forming biofilms, recent studies have also revealed that *E. faecalis* is able to survive inside host cells. They achieve this by developing mechanisms to resist phagosome acidification, thus promoting their intracellular survival in poly morphonuclear leucocytes and macrophages.^14,15^ These professional phagocytes now act as “Trojan Horses”, providing a reservoir of *E. faecalis* for further infections. Intracellular *E. faecalis* is protected from antibiotics, especially hydrophilic Amp, because of the barrier posed by the host cellular membrane. In fact, biofilm-related and intracellular infections can occur concurrently, thus greatly reducing the efficacy of any treatment. Taken together, there is a need to develop new antibiotic delivery strategies against *E. faecalis* infections at every juncture of pathogenesis, targeting both extracellular and intracellular states.

The idea of antibiotic delivery using nanoparticles has been largely explored and showed great potential against certain biofilm-mediated and/or intracellular pathogenic infections.^16−18^ For example, when encapsulated in cationic nanostructured lipid carriers, oxacillin showed synergistic activity against methicillin-resistant *Staphylococcus aureus* (MRSA) for cutaneous infections.^19^ Among the various nanoparticle-based delivery systems, Lipid-Polymer hybrid Nanoparticles (LPNs) possess great advantages in situations for both biofilm-mediated and intracellular infections.^20^ LPNs are nontoxic, programmable, and could be fabricated through a simple and economical approach. The cationic lipid surface not only promotes colloidal stability but prevents premature release of antibiotics. The positive surface charge also provides higher binding affinity toward both planktonic bacteria and biofilms, providing localized delivery and minimizing systemic exposure, whereas the polymeric core contributes to the sustained antibiotic release to bacterial biofilms shown in our previous study.^21^ On the basis of our conjecture from previous studies, LPNs can be engulfed by the professional phagocytes through phagocytosis, which is the same transport pathway as pathogens. This allows the loaded antibiotic to be delivered into the infected cells, thereby enhancing its penetration against intracellular pathogens.

Currently, there is no “holistic” infection model that monitors the concurrence of intracellular infections and high MOI biofilm infections for *E. faecalis* in phagocytes. Phagocytic cells like macrophages cannot survive when incubated with a high MOI of biofilm bacteria *in vitro*, thus a low MOI of only 10 is often used in biofilm-macrophage studies.^30,31^ Herein, we established a surrogate phagocytic model, using *Tetrahymena pyriformis* coculture model to assess the bactericidal activity of ampicillin-loaded lipid polymer hybrid nanoparticles (Amp-LPNs) against *E. faecalis*; composing of planktonic, biofilm, and intracellular forms of bacteria in the model, with a high MOI of 10^4^ or 10^5^. The ciliated *T. pyriformis* is a unicellular model organism whose characteristics have been extensively studied, and it has been used as a test system for toxicological evaluations for more than 40 years.^22−24^ In this study, *T. pyriformis* has been selected as a systemic representative carrier of both biofilm and intracellular infections. *T. pyriformis* identifies microbes via chemotaxis and ingests bacteria through a phagocytic food vacuole, which greatly imitates the behavior of phagocytes (e.g., macrophages and neutrophils) in the human innate immune system.^32^ Besides, it has been shown in our study that part of the ingested bacteria will be expelled back to the environment, enabling this *E. faecalis* infected *T. pyriformis* coculture model to resemble the full infection cycle of chronic infections caused by aggregated intracellular pathogens.^34^ In this study, we developed and evaluated the LPN delivery system, loaded with Amp that would be taken up by a novel *ex vivo* model of the infected *T. pyriformis*, to deliver the antibiotic at the site of intracellular infection to elicit enhanced antimicrobial effects and demonstrated the potential prophylactic effectiveness of this approach to alleviate the host cells’ burden of both biofilm and intracellular pathogens.

## Results and Discussion

### PLGA/DOTAP Nanoparticle Fabrication and Characterization

Ampicillin is a typical β-lactam antibiotic which rapidly exerts bactericidal effects against *E. faecalis in vitro*. However, the opportunistic pathogen *E. faecalis* resists phagosome acidification and autophagy after being phagocytosed by macrophages and survives intracellularly in the host cells.^14^ The lipophilic nature of cell membrane impedes the penetration of hydrophilic Amp into the intracellular space. Therefore, enabling the accessibility and continuous exposure of antibiotics to *E. faecalis* is essential. In this study, we demonstrated the ability of the LPN-based antibiotic delivery system in achieving this goal.

**Figure 1:**
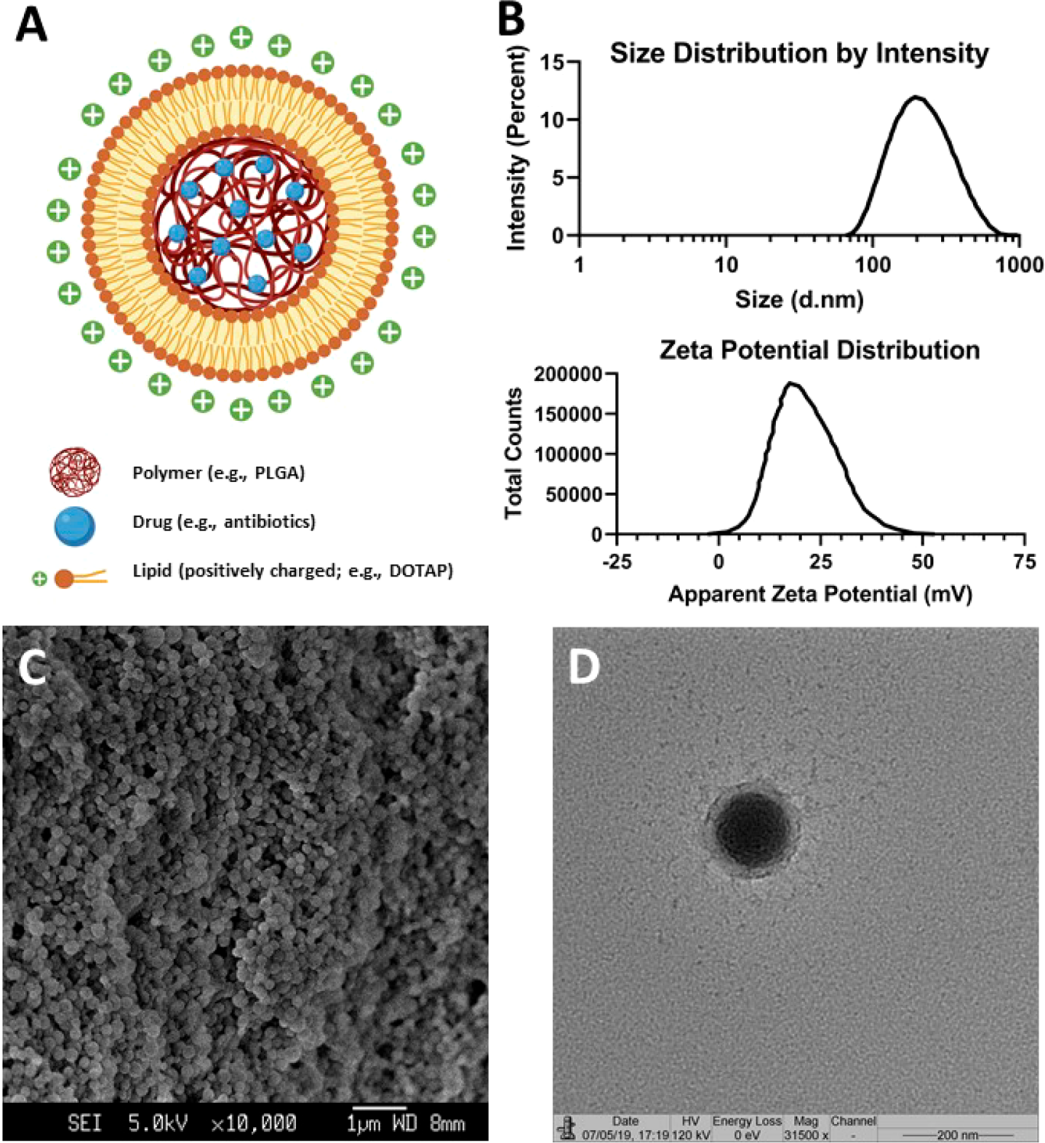
Amp-LPN fabrication and characterization. (A) A schematic diagram of an Amp-LPN (created with BioRender.com). (B) The size distribution and ζ-potential of Amp-LPN nanoparticles. (C) SEM (Scale bar = 1 μm) and (D) TEM images of Amp-LPN nanoparticles (Scale bar = 200 nm).

In this report, we successfully fabricated Amp-LPNs with a core–shell structure using a combination approach of emulsion-solvent-evaporation and lipid thin-film rehydration, with the molecular structure illustrated in Figure 1A. Around 20 μg ampicillin was encapsulated in 1 mg particles. This formulation is capable of achieving sustained release, with only 10% of the drug released under the experimental conditions on the fifth day (Figure S3A). The average diameter of Amp-LPNs was 193.8 ± 1.908 nm, with a narrow unimodal distribution (PDI = 0.166 ± 0.003) (Figure 1B and Figure S5). These particles were spherical in shape as observed under SEM (Figure 1C). With DOTAP as the surface cationic lipid coating, Amp-LPNs exhibited a positively charged ζ potential of 20.5 ± 0.566 mV, suggesting the cationic DOTAP lipid molecules were successfully coated onto the surface of PLGA nanoparticles (Figure 1B and Figure S6). This core–shell structure was further confirmed using TEM (Figure 1D). Meanwhile, the size, distribution and ζ potential did not change significantly, suggesting this formulation was stable (Figure S3B,C,D).

### Establishment of an *E. faecalis-T. pyriformis* Infection Model

We have previously reported that antibiotic-loaded LPNs hold the potential to significantly reduce the viability of planktonic and biofilm Gram-positive and Gram-negative bacteria strains.^21^ In this paper, the potential of LPNs as an antibiotic carrier against an intracellular and/or biofilm pathogenic infection model was investigated. Herein, an *E. faecalis*-infected phagocytic protozoa cell, *T. pyriformis*, was therefore chosen and developed as a model system. Protozoa have been used as an alternative to the mammalian phagocytes due to their ability to use chemotaxis to trace bacteria and ingest them through phagocytosis. Besides, the strategies used by bacteria to survive the natural predation are also used in escaping from phagocytes,^33^ for example, *T. pyriformis* has previously been used as a model organism to investigate how Listeriolysin O (LLO), a major virulence factor in *Listeria monocytogenes* infections in mammals, promoted bacterial survival under protozoa’s predation.^25,34^ Lastly, the choice of *T. pyriformis* allows us to overburden the cells with a much higher MOI over macrophages. On the basis of this, an *E. faecalis-T. pyriformis* coculture model was established to assess antibacterial activities of Amp-LPNs intimating both acute and chronic infections.

**Figure 2:**
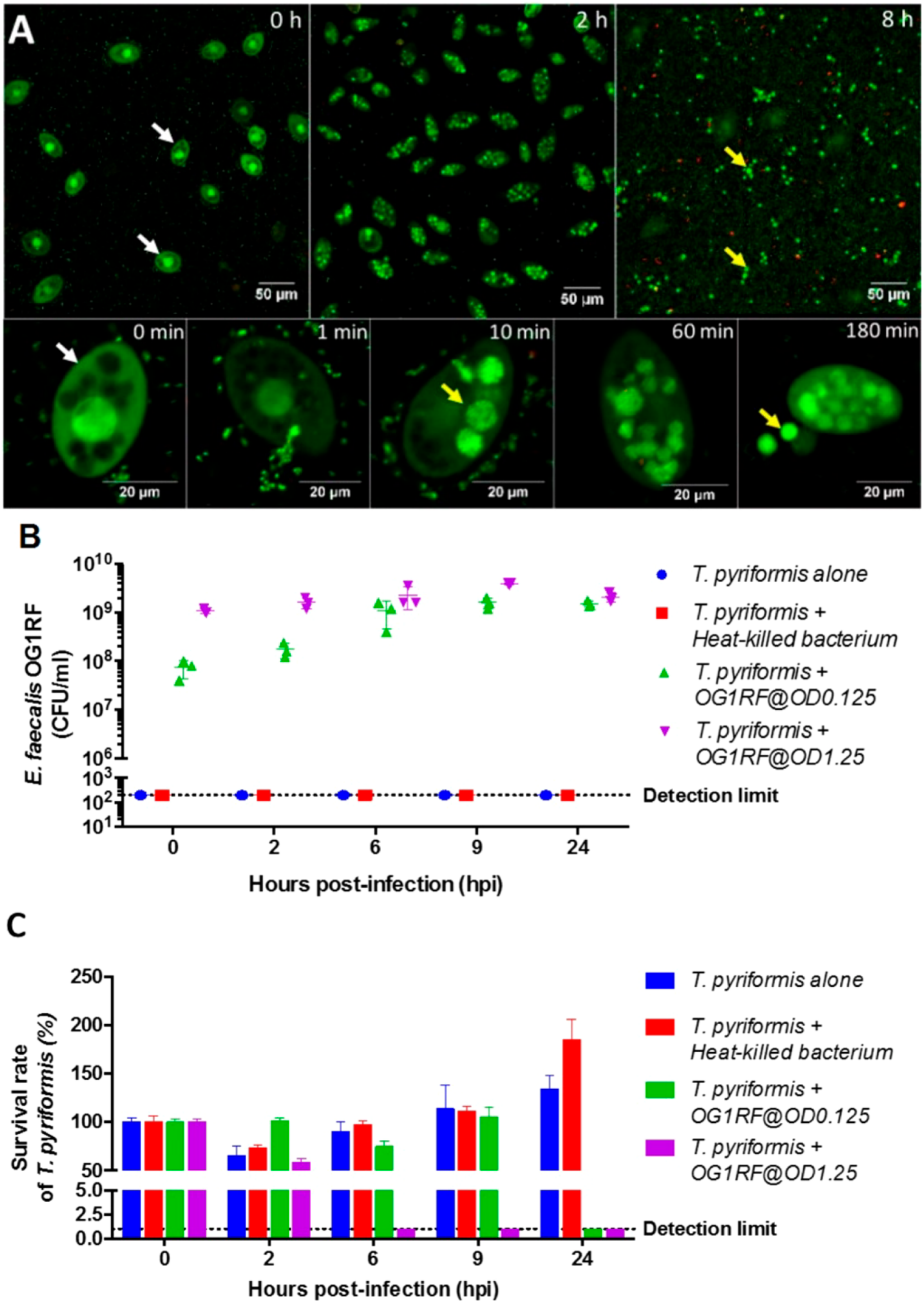
*E. faecalis* and *T. pyriformis* coculture model. (A) The infection cycle of the host *T. pyriformis* by *E. faecalis*. The *E. faecalis-T. pyriformis* coculture was sampled over time and labeled using bacterial Live/Dead stain (i.e., syto9/propidium iodide) prior to CLSM imaging. (B) The *E. faecalis* cell count (CFU/mL) enumerated on TSB agar, and (C) the *T. pyriformis* survival rate during the course of infection (Mean ± SD, *n* = 6).

Before application of this model to evaluate the bactericidal effects of Amp-LPNs, the lifestyle of this pair of protozoan predator and bacterial prey was investigated. We found *T. pyriformis* (Figure 2A, indicated by white arrows) engulfed *E. faecalis* immediately after being cocultured and all the food vacuoles of *T. pyriformis* were saturated by *E. faecalis* after 2 h. Interestingly, these intracellular *E. faecalis* aggregates (yellow arrows) were released into the extracellular environment from 3 h onward.

*T. pyriformis* was incubated together with either heat-killed *E. faecalis* or live *E. faecalis* with a starting CFU count of 10^8^ CFU/mL (OD 0.125) or 10^9^ CFU/mL (OD 1.25). Live bacteria kept proliferating in the presence of *T. pyriformis* until reaching the cell density limit (∼10^9^ CFU/mL). By enumerating *T. pyriformis* and the CFU of *E. faecalis* after coincubation (Figure 2B,C), we first confirmed that *T. pyriformis* as a predator was able to utilize *E. faecalis* as a nutrient source. It was observed that *T. pyriformis* that were feeding on heat-killed bacteria proliferated to over 150% of the starting count. Protozoa that consumed heat-killed bacteria could achieve higher growth compared to just liquid nutrient medium. However, when *T. pyriformis* was incubated with live *E. faecalis*, there was a competition between the predator and prey, and the bacterial density determined the health status of protozoa. For a starting bacterial concentration of 10^8^ CFU/mL (OD 0.125), *T. pyriformis* was able to maintain its viability up to 9 h. As the bacteria kept proliferating, *T. pyriformis* was overwhelmed, with no survivors found after 24 h. When incubated with *E. faecalis* at the saturation cell density (10^9^ CFU/mL, OD 1.25), *T. pyriformis* ceased after 2 h with no survival after 6 h. Thus, a bacterial density of 10^8^ CFU/mL (OD 0.125) was chosen for subsequent experiments, providing a more reasonable and longer operable time frame to explore the performance of Amp-LPNs in different interventions in this coculture model.

### Amp-LPNs Can Abrogate *E. faecalis* Infections during Early Intervention

**Figure 3:**
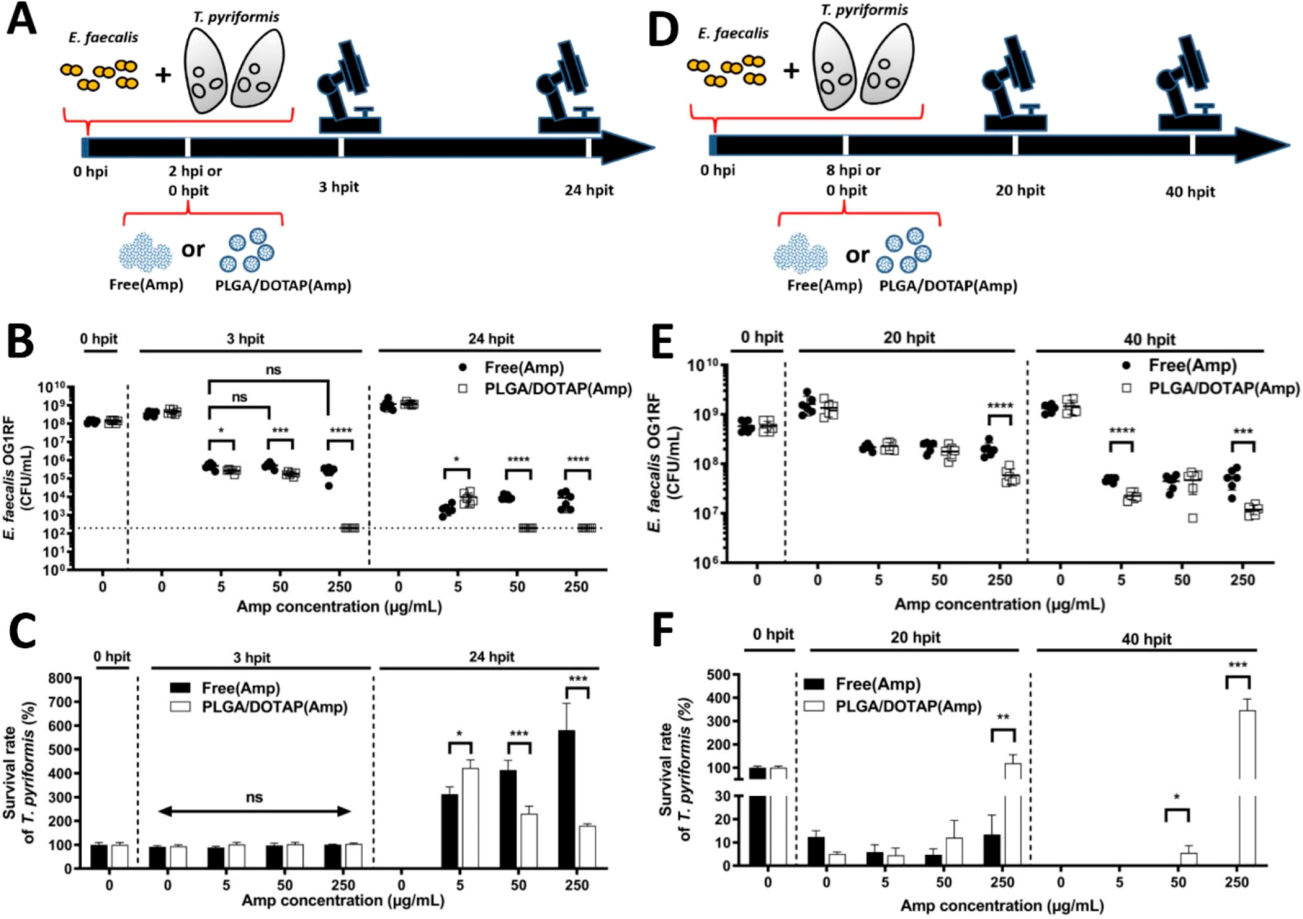
Amp-LPN mediates the clearance of *E. faecalis*. (A) The *T. pyriformis* was infected by *E. faecalis* at OD 0.125 2 h prior to the free Amp and Amp-LPN treatments (early intervention). (B) The *E. faecalis* cell count (CFU/mL) and (C) the *T. pyriformis* survival count were assessed 3 and 24 h post infection treatment (hpit). (D) The *T. pyriformis* was infected by *E. faecalis* at OD 0.125 8 h prior to the free Amp and Amp-LPN treatments (late intervention). (E) The *E. faecalis* cell count (CFU/mL) and (F) the *T. pyriformis* survival count were assessed 20 and 40 h postinfection. Multiple *t* tests with Holm-Sidak method were performed to investigate the effect of LPN encapsulation. One-way ANOVA test with Dunnett’s multiple comparison was performed for free Amp groups at 3 hpit. The statistical differences are indicated as follows: * *P* < 0.05, ** *P* < 0.01, *** *P* < 0.001, **** *P* < 0.0001, and ns stands for nonsignificant. The results shown are representatives of three independent experiments (Mean ± SD, *n* = 6).

Moving on to examining the antimicrobial activity of the Amp-LPNs to suppress the viability of different forms of *E. faecalis* in infected *T. pyriformis*, we first validated that blank LPNs did not possess any toxicity toward *E. faecalis* and *T. pyriformis* (Figure S4). Referring back to Figure 2A,C, at the time point of 2 h after infection, the food vacuoles of *T. pyriformis* were saturated by *E. faecalis* and most *T. pyriformis* cells remained viable. This time point was chosen as an early intervention time point for the antimicrobial assessment of Amp-LPNs to resemble an acute infection (Figure 3A).

Significant reduction in bacterial count was found in all groups treated with either free or LPN formulations at all Amp concentrations, confirming the antimicrobial activity of Amp against *E. faecalis* (Figure 3B). In all cases, Amp-LPNs achieved a better killing effect than the free formulation using the same concentrations of Amp. After 3 hpit, complete bacterial clearance (below limit of detection) was shown at an Amp-LPN concentration of 250 μg/mL, achieving >6 logs reduction compared with the control, while the free formation managed to reduce approximately 3 logs of bacterial cells under the same concentration. Increasing the dose of free Amp from 5 to 250 μg/mL did not present any noticeable improvements on antimicrobial activity, which might be limited by the penetration problem of free β-lactams or the short intracellular retention time. Further increase of free Amp to 250 μg/mL only showed a slight decrease in the CFU count (<1 log) compared with 50 μg/mL. At the 24 hpit assessment, complete elimination (below the limit detection) of *E. faecalis* was achieved even at an Amp-LPN concentration of 50 μg/mL, but not for the free drug even at 250 μg/mL. This suggests that Amp-LPNs provided enhanced delivery of β-lactam drugs, which permits complete clearance of *E. faecalis* during early intervention.

No difference was detected for the protozoa count for both free and LPN formulations at all Amp concentrations after 3 h treatment (Figure 3C). After 24 h antibiotic treatment, protozoa multiplied to different extents, due to the alleviated burden from *E. faecalis*. In general, the survived protozoa in all groups were over 200% compared to time 0, suggesting bacterial load was controlled by both free Amp/Amp-LPNs in synergy with protozoa phagocytosis. For groups treated with free Amp, the number of the final *T. pyriformis* count increased 350–600% after 24 hpit and followed a positive concentration-dependent manner in the concentration range of 5–250 μg/mL. We speculated that a higher concentration of free Amp could act more rapidly on the bacteria that were expelled from the protozoa into the extracellular environment, which could be consumed by *T. pyriformis* as a source of nutrients for proliferation. Interestingly, for groups treated with Amp-LPNs, a higher treatment concentration did not lead to a higher survival rate of *T. pyriformis* at the time point of 24 hpit. A negative correlation between the number of survived protozoa and the concentration of Amp-LPNs was observed (despite a significant overall growth of protozoa compared to 0 hpit). As neither blank LPNs nor ampicillin had any toxicity on protozoa (Figure S4), it was postulated that this observation was due to the more efficient killing of *E. faecalis* at higher Amp-LPN concentrations (Figure 3B). The potency of Amp-LPNs resulted in overall fewer inactivated bacteria as a food source in this coculture. Compared with Figure 2C, it was noted that *T. pyriformis* receiving 250 μg/mL Amp-LPNs treatment proliferated to the same extent as those exposed to heat-killed bacteria (∼200%), suggesting bacteria in the 250 μg/mL Amp-LPNs treatment were mostly inactivated.

### Amp-LPNs Can Protect Protozoa from Bacterial Killing during Late Intervention

Considering that the engulfed bacteria would be expelled back to the environment after 3 h of coincubation and cause reinfection, and *T. pyriformis* was able to maintain its viability up to 9 h (Figure 2C), the time point of 8 h after infection was selected as the time point for the late intervention. This was to investigate whether Amp-LPNs could protect *T. pyriformis* in the event of chronic infection (Figure 3D).

Similarly as seen in Figure 3B, reduced CFU numbers of *E. faecalis* were observed in all groups receiving antimicrobial therapy in the late intervention (Figure 3E). At the highest concentration of Amp examined, i.e., 250 μg/mL, the LPN formulation exhibited a statistically significant difference in antimicrobial activity compared with the free formulation at the same concentration in both 20 hpit and 40 hpit. Amp-LPNs at 250 μg/mL were able to reduce the CFU counts from 10^9^ to 10^8^ CFU/mL in the first 20 h and another one log reduction in the 40 hpit assessment. Meanwhile, with the increased concentrations of Amp, especially in LPNs, the survival of *T. pyriformis* was significantly improved in the following 20 h (Figure 3F). All free Amp groups failed to save *T. pyriformis* in the 40 h incubation time while the Amp-LPNs (250 μg/mL) presented the highest survival rate, approaching 400% at the 40 h postinfection time point (Figure 3F). We speculated this observation was due to the presence of biofilms, which blocked the free Amp approaching bacteria and thus prevented the reduction in bacterial numbers for late intervention. However, the LPNs with positively charged surface provided better affinity to bind bacterial cells and biofilm, and the release of Amp from LPNs provided sustained antibacterial effects. This also proved that the LPNs were nontoxic toward *T. pyriformis* cells even at high concentrations.

**Figure 4:**
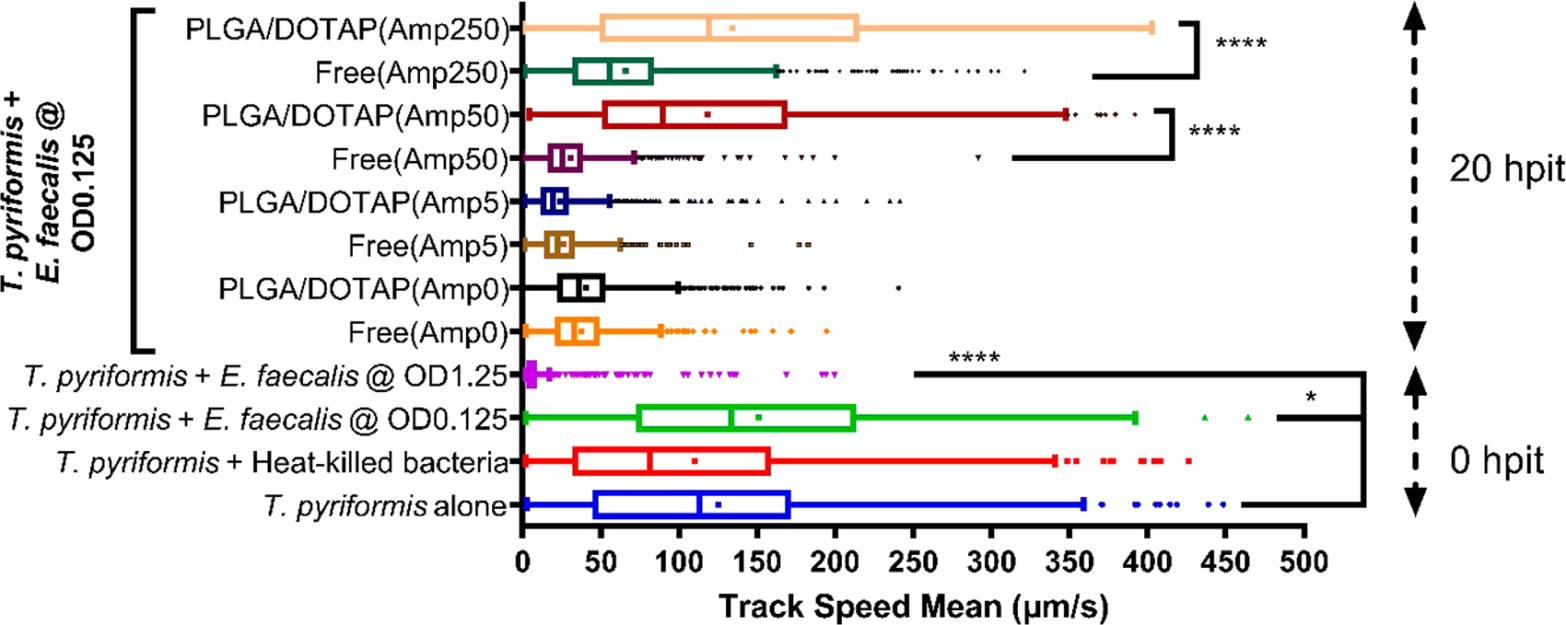
Motility of *T. pyriformis* determined as the track speed mean was assessed 0 and 20 h postinfection treatment. Bar in the middle represents the median, and dot represents the mean. For the downmost 4 groups, nonparametric Kruskal–Wallis test was performed in comparison with the group of “*T. pyriformis* alone”, respectively. For the other 8 groups at 20 hpit, nonparametric Kruskal–Wallis test with multiple comparisons was performed to investigate the effect of LPN encapsulation. The statistical differences are indicated as follows: * *P* < 0.05, **** *P* < 0.0001.

To further confirm the viabilities and activities of the *T. pyriformis* cells after being infected and/or treated, their motilities were assessed at 0 hpit (8 hpi) and 20 hpit, the same condition that we used for the late intervention (Figure 4). After 8 h coincubation with *E. faecalis* (i.e., 0 hpit), *T. pyriformis* remained alive and active except the ones subjected to 10^9^ CFU/mL bacteria, which was in agreement with our previous observation shown in Figure 2C. At 20 hpit, for all groups receiving free Amp, protozoa motility was largely compromised compared to their active status. For groups treated with high concentrations of Amp-LPNs (50 and 250 μg/mL), motility was mostly preserved. Together, these results demonstrated that Amp-LPNs could effectively protect *T. pyriformis* from *E. faecalis* killing during the late intervention and sustain their activity. This demonstrated another advantage of the protozoa model, as we can simply measure the fitness of the protozoa (i.e., motility) under different conditions.

From the above-observed results, it can be concluded that Amp-LPNs significantly enhanced the antibacterial activity of Amp in early and late interventions, compared to the unformulated free drug. Since this protozoa coculture model was established to imitate the phagocytic behavior of macrophages, it correlates well with the macrophage uptake findings that the improved uptake of antibiotic-loaded nanoparticles by macrophages translates to an increased intracellular concentration of the loaded antibiotics and consequently, better bacterial killing. This is in agreement with some recent studies that have demonstrated improved synergistic effects of antibiotics-loaded nanoparticles compared to free antibiotics at corresponding concentrations, suggesting that the increase in nanoparticle uptake by macrophages translates to the enhanced antibacterial activity against intracellular infections.^26−28^

### Prophylactic Treatment Using Amp-LPNs Enables *E. faecalis* Eradication by *T. pyriformis*

**Figure 5:**
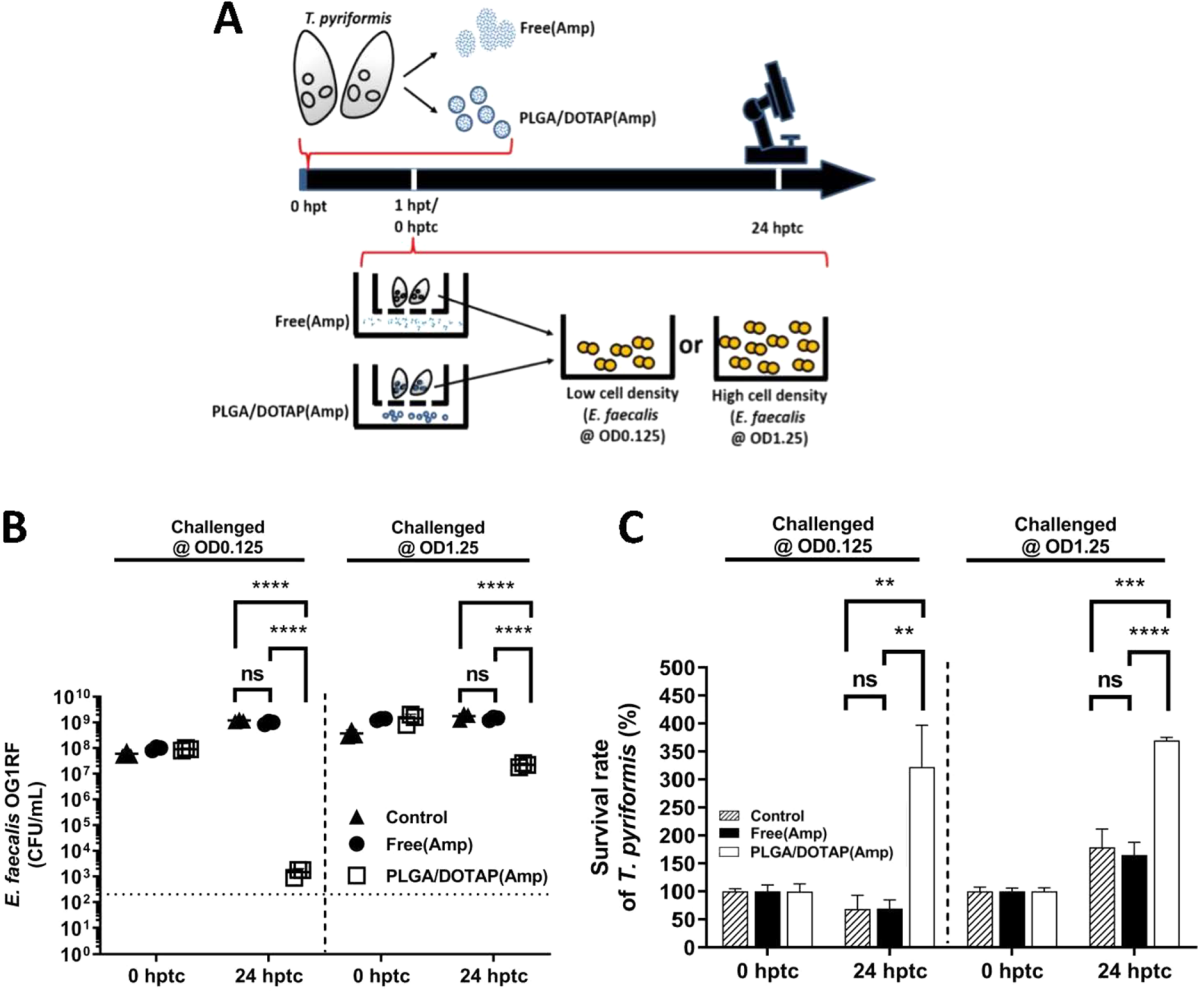
Prophylactic treatment using Amp-LPNs enables *E. faecalis* eradication by *T. pyriformis*. (A) The experimental setup. *T. pyriformis* was treated by ampicillin (Amp) 1 h prior to the *E. faecalis* challenge at OD 0.125 and OD 1.25. Excess extracellular Amp (either as free or in LPN formulation) was filtered and the *T. pyriformis* culture was washed five times in an eight-micrometer filter well. (B) The *E. faecalis* cell count (CFU/mL) and (C) the *T. pyriformis* survival count were assessed 24 h post-treatment challenge (hptc). One-way ANOVA comparisons corrected using the Tukey method where statistical differences are indicated as follows: ** *P* < 0.01, *** *P* < 0.001, **** *P* < 0.0001 and ns stands for nonsignificant. The results shown are representatives of three independent experiments (Mean ± SD, *n* = 6).

Next, based on the nature of *T. pyriformis*, we hypothesized that the Amp-LPNs taken by protozoa may provide a protective window against subsequent *E. faecalis* infection in *T. pyriformis* cells. To investigate our hypothesis, *T. pyriformis* was treated prophylactically with either free Amp or Amp-LPNs at 250 μg/mL 1 h prior to bacterial challenge. The pretreated *T. pyriformis* cells were filtered to remove excess extracellular Amp or Amp-LPNs and were subsequently infected with *E. faecalis*. The recovered viable bacteria and survived *T. pyriformis* cells were monitored for the subsequent 24 h (Figure 5A). In the following experiments, it was clearly observed that prophylactical free Amp in *T. pyriformis* had no inhibitory effects on intracellular *E. faecalis* (Figure 5B), and therefore it could not provide protection for protozoa from the subsequent infection (Figure 5C), which would suggest that Amp in its free form is not accumulating in *T. pyriformis* or it is rapidly excreted from *T. pyriformis*, although this would require further analysis. In contrast, a 1 h pretreatment of nanoparticles entrapped Amp elicited significant differences in bacteria eliminating and protozoa survival toward subsequent infections. It revealed those *T. pyriformis* cells, pretreated with Amp-LPNs, demonstrated significant CFU reduction (Figure 5B) and protozoa survival (Figure 5C) after 24 h incubation with *E. faecalis*, especially in the low bacterial density group. These results suggested that the controlled release effect of the nanoparticle formulation provided a prophylactic window against bacterial infections, which is in agreement with one of our previous studies, in which we observed the similar protective ability of gentamicin-loaded nanoparticles in the body of Galleria larvae against subsequent *Klebsiella pneumoniae* infection.^29^

### Mechanisms of Amp-LPNs-Mediated *E. faecalis* Eradication

**Figure 6:**
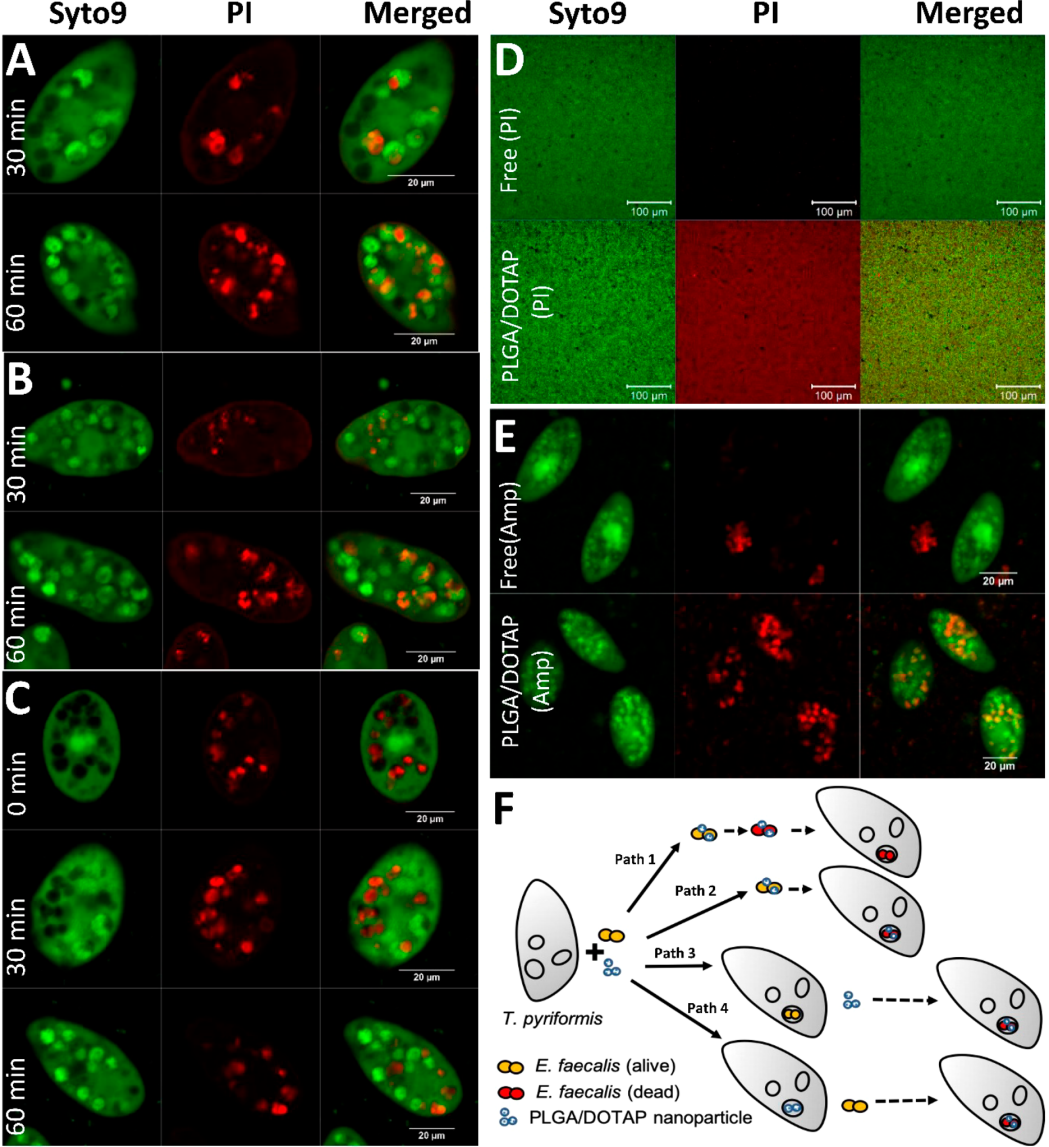
Proposed mechanism(s) of LPNs-mediated *E. faecalis* eradication. (A) Uptake of LPNs-PI cocultured with *E. faecalis* by *T. pyriformis*. (B) Uptake of LPNs-PI by *T. pyriformis* preinfected by *E. faecalis*. (C) Uptake of LPNs (PI) by *T. pyriformis* followed by *E. faecalis* challenge. (D) Labeling of *E. faecalis* biofilms by free PI or LPNs-PI. (E) Live/Dead staining (i.e., syto9 and PI) of *T. pyriformis* culture infected by *E. faecalis* after treatment using free Amp or Amp-LPNs. (F) Proposed mechanism(s) for LPNs-mediated *E. faecalis* eradication. Syto9 was used as a viable label for *T. pyriformis* and *E. faecalis* (A–E). Scale bars: 20 μm (A–C and E), 100 μm. (D) PI: propidium iodide.

From the discussion above, it can be concluded that Amp-LPNs not only significantly enhanced the antibacterial activity of Amp in early and late interventions but also provided prophylactic effects against subsequent infections. However, the results until this point were presented as a total killing effect, regardless of whether *E. faecalis* was in planktonic, intracellular, and biofilm form. To further characterize the antimicrobial activity of LPNs with regard to different forms of bacteria, a series of confocal microscopy experiments were conducted, focusing on the ability of Amp-LPNs to eradicate intracellular and biofilm form of bacteria, which are the hard nuts in current antimicrobial therapy. To investigate the mechanism of intracellular killing, first, the coincubated *E. faecalis* with PI-loaded LPNs were observed in the food vacuoles of *T. pyriformis* (Figure 6A), suggesting the cocultured bacteria and nanoparticles could both be taken up by the protozoa in the same food vacuole. We speculated this could be a possible mechanism that *E. faecalis* would be inactivated by the Amp-LPNs either before or after taken up by the protozoa. In order to further confirm this, we infected protozoa with *E. faecalis*, with subsequent labeling using PI-loaded LPNs. The cocultured bacteria and LPNs were also observed in protozoa (Figure 6B), indicating the infected *T. pyriformis* were still able to take up the nanoparticles. These LPNs could possibly enter the protozoa through phagocytosis or membrane fusion to eradicate the intracellular bacteria, although this required further investigations. Figure 6C demonstrated a reverse way where *T. pyriformis* was preincubated with PI-loaded LPNs and then infected with *E. faecalis*. It suggested that LPN-harboring protozoa were capable of taking up *E. faecalis* as well, and these Amp-LPNs were able to release antibiotic to kill bacteria intracellularly. The intracellular antimicrobial effects of Amp-LPNs were further confirmed with live/dead staining of *E. faecalis* (Figure 6E). Moving on to the ability of LPNs to penetrate the biofilm-embedded bacteria, *E. faecalis* biofilms were treated with either free or LPN-encapsulated PI to show the penetration effect. Almost an equal ratio of bacterial cells was labeled with PI in the biofilm after LPN treatment, indicating the LPNs bound to *E. faecalis* OG1RF biofilm with high affinity, which could account for their superior biofilm killing ability (Figure 6D). Taking these observations together and combining them with our previously obtained results, we hereby proposed four possible mechanisms of Amp-LPNs mediated *E. faecalis* eradication (Figure 6F). Amp-LPNs could work in the extracellular and/or intracellular space in this infection model. Having excellent binding affinity toward bacteria, Amp-LPNs could form a complex with *E. faecalis*, which is then taken up by *T. pyriformis*. In this way, *E. faecalis* can be inactivated in the extracellular space and then engulfed by protozoa as nutrients (path 1) or conversely, first taken up by protozoa and then inactivated intracellularly (path 2). From the view of postinfection treatment, Amp-LPNs could be taken up by infected protozoa to achieve an intracellular antimicrobial therapeutic effect (path 3). Furthermore, Amp-LPNs could also work as a prophylactic treatment to protect the host from subsequent infections (path 4).

## Conclusion

In this study, we fabricated an ampicillin encapsulated lipid-polymer hybrid nanoparticle system which hybridizes the properties of liposomal and polymeric systems. For the first time, we demonstrated the utility of an *E. faecalis* and *T. pyriformis* coculture model as a surrogate phagocytic model to assess the anti-intracellular bacteria and antibiofilm activity of antibiotic-loaded LPNs. The results support the utility of this nanoantibiotic technology for the treatment of intracellular *E. faecalis*. It is evident that the LPN is capable of improving the therapeutic efficacy of Amp to combat *E. faecalis* in both early and late interventions, and also provides significant prophylactic effectiveness for *T. pyriformis* cells. Although protozoa may, in some cases, not completely mimic tissue- or whole-animal-level processes, they are extremely flexible, and their use should be embraced. It is also believed and expected that with further investigations into the physiochemical properties of antibiotic-loaded LPNs and a deeper understanding of the relationship between the host and invading pathogens, the LPN system can be endorsed with further enhancing antimicrobial effects toward different bacteria at different severities of infection.

## Methods

### Materials

DOTAP (1,2-dioleoyl-3-trimethy-lammonium-propane, chloride salt) was purchased from Avanti Polar Lipids (Alabaster, AL, USA). PLGA (502H, MW 7000–17000), ampicillin, propidium iodide (Invitrogen), SYTO 9 Green Fluorescent Stain (Invitrogen), and other chemicals at analytical grade were supplied by Sigma-Aldrich Singapore, unless otherwise specified.

### Bacterial Strain and Protozoa Culture

*E. faecalis* (OG1RF) was routinely maintained in tryptic soy broth (TSB) or agar (Oxoid, UK) at 37 °C for 24 h. Heat-killed *E. faecalis* was prepared by growing it overnight in TSB at 37 °C and adjusting it to OD_600_ = ∼ 1.25 (i.e., 1–2 × 10^9^ CFU/mL) before transferring it to a water bath at 65 °C for 2 h. The viability of the heat-killed *E. faecalis* was tested by its plating on TSA (1.5% w/v) (Oxoid, UK) at 37 °C for 48 h. Heat-killed *E. faecalis* stocks were stored at −20 °C. *E. faecalis* biofilm was established as described previously.^21^ Briefly, 200 μL of bacterial culture was seeded in a 96-well plate at a concentration of 8 × 10^6^ cells/mL for 8 h at 37 °C. The spent medium was removed, and the biofilm was washed with TSB gently to remove the planktonic bacteria cells.

*T. pyriformis* (CCAP 1630/1W, CCAP, UK) was maintained axenically in a 15 mL sterile peptone-yeast-glucose medium (20 g/L of proteose peptone and 1 g/L of yeast extract) supplemented with 0.1 M sterile-filtered glucose in a 25 cm^2^ tissue culture treated flask (Falcon, Fisher Scientific, USA) with ventilated caps and incubated statically at room temperature. Prior to experiments, 500 μL of axenic *T. pyriformis* was passaged in 15 mL pf TSB and incubated with shaking (50 rpm) at room temperature for 72 h. This process ensured that *T. pyriformis* was acclimatized to the change in the growth media and a high abundance of *T. pyriformis* after 72 h. *T. pyriformis* was quantified by removing 10 μL aliquots in triplicates from the axenic culture and counting them using light microscopy (Primo Star, Carl Zeiss, Germany).

### Fabrication of Ampicillin-Loaded Lipid-Polymer Nanoparticles (Amp-LPNs)

A combination approach of emulsion-solvent-evaporation and lipid thin-film rehydration was adopted for the development of the Amp-LPNs. Briefly, 10 mg of Amp was dissolved in 0.5 mL of 1% aqueous poly(vinyl alcohol) (PVA) solution (w/v, in 0.95% MES buffer, pH 7), followed by addition of 2 mL of dichloromethane (DCM) containing 60 mg of PLGA. The primary emulsion was obtained by probe-sonicating the mixture at 50 W for 12 cycles in pulse mode (3 s on, 2 s off). Subsequently, the primary emulsion was added dropwise into 10 mL of aqueous PVA solution via a 25G needle. The mixture was sonicated for another 18 sonication cycles, as before, to obtain a water in oil in water formulation. The nanoparticle suspension was stirred for 4 h to evaporate DCM and then washed twice by centrifugation-resuspension cycles (at 20000 *g*, 20 min, 4 °C) in deionized water. The nanoparticles were resuspended in deionized water for the following lipid coating. The DOTAP thin-film was prepared by solvent evaporation of a DOTAP-containing DCM in a rotary evaporator. The core–shell structure was obtained through direct hydration of the DOTAP thin-film within the nanoparticle suspension by tender sonication. Finally, the nanoparticle suspension was washed again, and the pellet was freeze-dried and kept at −20 °C.

### Characterization of Amp-LPNs

Triplicate LPN batches were diluted in deionized H_2_O and characterized by Zetasizer (Malvern Nano ZS) measurements for mean particle size, polydispersity index (PDI), and ζ-potential. For Field Emission Scanning Electron Microscopy (FESEM, JEOL-6340F), small droplets (10 μL, 5 mg/mL) of Amp-LPNs were dried and sputter-coated with platinum on metal stubs and visualized. Transmission Electron Microscopy (TEM, Carl Zeiss Libra 120 Plus) was applied to observe the core–shell structure of LPNs. TEM samples were prepared by the addition of an Amp-LPN suspension onto a hydrophilic Formvar-coated copper grid for 3 min, followed by uranyl acetate staining.

Drug loading was calculated based on analyzing residual ampicillin in the supernatants obtained during nanoparticle washing. The concentration of ampicillin was determined by measuring the absorbance at λ = 268 nm using a Cary UV–vis spectrophotometer (Agilent Technology, USA).^35^ Calibration curves were established using known concentrations of ampicillin dissolved in the supernatant of blank-LPNs. In this way, interferences were taken into consideration at each concentration.

The release of ampicillin from the LPNs was assessed using 2 mL of LPNs (25 mg/mL) in PBS buffer (pH 7), which was injected into a Slide-A-Lyzer Dialysis Cassette 7000 MW (Thermo Scientific, UK). The cassettes were then placed into an 18 mL reservoir of PBS buffer in an incubator at 37 °C, with shaking at 150 rpm. At predetermined time points, 5 mL samples were removed from the reservoir and replaced with 5 mL fresh PBS buffer. The ampicillin content in samples was quantified by comparison to standards containing known amounts of ampicillin in PBS buffer. Stability studies were performed under the parameters of release study and up to 5 days in PBS with shaking (120 rpm) at 37 °C. LPN batches were made in triplicate, and Zetasizer analyses (size, PDI, and ζ-potential) were performed in duplicate per batch.

### *E. faecalis* and Protozoa Coculture Model

A coculture model consisting of *E. faecalis* and *T. pyriformis* was established. Briefly, a single *E. faecalis* colony was inoculated in 10 mL of TSB for 18 h at 37 °C while shaking at 150 rpm. The bacterial cells were washed once with 1X phosphate buffer saline (PBS) and diluted to a defined optical density (OD_600_) of 0.125 or 1.25 in TSB. *T. pyriformis* was cultured in TSB and quantified as detailed in Section 2.2. To establish the bacteria-protozoa coculture model, *T. pyriformis* was added to *E. faecalis* culture of either OD_600_ = 0.125 or 1.25 in TSB to achieve a final protozoa concentration of 10^4^ cells/mL. Similarly, *T. pyriformis* was supplemented with heat-killed *E. faecalis* to establish a heat-killed bacteria-protozoa control. These cocultures were aliquoted in triplicates into a 24-well microtiter plate and incubated with shaking (50 rpm) at room temperature. The antimicrobial effect of Amp-LPNs on *E. faecalis* was investigated by either early or late introduction of Amp-LPNs to the bacteria-protozoa coculture. Early intervention involved the addition of either Amp-LPNs or free Amp to 2 h postinfection (hpi) cocultures with continued incubation with shaking (50 rpm) at room temperature. The viability of *E. faecalis* after Amp-LPNs or free Amp treatment was determined by serially diluting aliquots of cocultures in 1X PBS at 3 and 24 h postinfection treatment (hpit) before plating on TSA plates for overnight incubation at 37 °C. Overnight colonies of *E. faecalis* were counted and the viability of *E. faecalis* was expressed as colony forming units/mL (CFU/mL). The survival rate of *T. pyriformis* was also determined at 3 and 24 hpit by enumerating them using light microscopy as detailed in Section 2.2. The late intervention involved the addition of either Amp-LPNs or free Amp to 8 hpi cocultures with continued incubation with shaking (50 rpm) at room temperature. The viability of *E. faecalis* and the survival rate of *T. pyriformis* were determined at 20 hpit and 40 hpit.

The prophylactic potential of Amp-LPNs was investigated by treating *T. pyriformis* with either free Amp or Amp-LPNs at 250 μg/mL for 1 h prior to a high and low concentration bacterial challenge. Residual free Amp or Amp-LPNs in the culture media after treatment for 1 h was removed via diffusion by fresh medium exchange using an eight-micrometre filter. Briefly, an eight-micrometre membrane insert filter was placed in a well of a six-well microtiter plate containing one milliliter of fresh TSB for medium exchange. One milliliter of the treated *T. pyriformis* culture was added to the eight-micrometre filter and allowed for free Amp or Amp-LPNs to diffuse into fresh TSB for 5 min. The filter containing the protozoan culture was subsequently transferred to the next well containing one milliliter of fresh TSB. This process was repeated 5 times to ensure that no residual extracellular Amp or Amp-LPNs remained in the treated *T. pyriformis* culture medium prior to the bacterial challenge. Thereafter, the treated and washed protozoa were subsequently challenged with OG1RF at OD 0.125 or OD 1.25 for 24 h with shaking (50 rpm) at room temperature. The effectiveness of Amp-LPNs or free Amp prophylaxis was determined by assessing the viability of *E. faecalis* and the survival rate of *T. pyriformis* as detailed above.

### Imaging and Image Analysis

Samples cultured under the same condition used in late intervention were imaged with a Zeiss AxioObserver Z1 inverted widefield microscope using 5X Objective (EC Plan-Neofluar) with 0.16 NA under brightfield illumination. For each imaging experiment, at least six fields per well were imaged by time lapse imaging for 30 s at maximum available camera speed (426 frames in total) with 1.4 ms exposure time. Image analysis and quantifications were carried out using both the open-source image analysis software ImageJ/Fiji and commercial Imaris v 9.0.2, Bitplane AG. Time lapse images were converted from original 12 Bit Carl Zeiss Image(czi) format to lossless TIFF series and pixel values were inverted (black to white pixel assignment) using the *Edit > Invert* command on ImageJ/FIJI (give the link of reference) and saved again as TIFF series. Consequently, Median projection type was applied using the ImageJ/FIJI Z projection > Median command and the corresponding image (Median projection) was subtracted from the original TIFF series to remove the nonmotile objects from further analysis. Subsequently, the TIFF series were loaded to Imaris v 9.0.2, Bitplane AG for further analysis. Individual *Tetrahymena* cell on time lapse images was detected using the ’Spots’ function on Imaris. The Thresh holding method was optimized to maximize the detection of individual cells, saved, and subsequently applied for all time lapse images during analysis. Time lapse images were grouped accordingly as per treatment methods. Quantifications and analysis statistics were exported as Comma Separated Value (csv) files for each parameter (ex: Track Speed Mean, Area, etc.) and were used for generating results.

### Fluorescent Dye Labeling to Reveal the Uptake of LPNs by *T. pyriformis*

To investigate how Amp-LPNs mediated *E. faecalis* eradication, propidium iodide (PI) was loaded in LPNs (LPNs-PI) and colocalization of LPNs-PI with *E. faecalis* in the presence of *T. pyriformis* was examined in three different scenarios: (1) LPNs-PI or free PI treatment of a cocultured *T. pyriformis* with *E. faecalis*, (2) *T. pyriformis* was preinfected with *E. faecalis* for 30 min prior to exposure to LPNs-PI or free PI, and (3) *T. pyriformis* was pre-exposed to LPNs-PI or free PI for 30 min prior to infection by *E. faecalis*. Similarly, the ability of LPNs-PI or free PI binding to *E. faecalis* biofilms was determined. For the biofilm study, a 24 h biofilm culture was pre-established on an 8-well glass chamber prior to exposure to the nanoparticles. In all cases, a total of 27.2 μM free PI or PI encapsulated in LPN (10 mg/mL) was applied and the cultures were incubated at room temperature in dark. All cultures were counterstained with Syto9 prior to examination under a confocal laser scanning microscope (CLSM) (Zeiss LSM 780, Carl Zeiss Singapore) at excitation/emission wavelengths of 480/500 nm and 490/635 nm for Syto9 and PI, respectively. Lastly, a *T. pyriformis* culture preinfected with *E. faecalis* for 2 h was treated with 250 μg/mL Amp-LPNs or free Amp for additional 3 h prior to labeling by Syto9/PI and visualization using a CLSM.

### Statistical Analysis

Results were analyzed with GraphPad Prism, version 8.4.3, GraphPad Software (San Diego, USA). As detailed in figure legends, ANOVAs, multiple *t* tests and nonparametric analyses with *P*-value corrections were conducted. Statistical significance critical values were defined as *P < 0.05, **P < 0.01, ***P < 0.001, and ****P < 0.0001.

## Supporting Information Available

Residual Amp activity in the filter well, after filtration and washing, which was assessed using the *E. faecalis* culture; residual Amp activity in a *T. pyriformis*-free control well, after filtration and washing, which was assessed using the *E. faecalis* culture; characteristics of Amp-LPN and Blank-LPN, including the release profile, size distribution, PDI, and ζ-potential; toxicity tests of blank LPNs on *E. faecalis* and protozoa; raw data report of size distribution and ζ-potential distribution by Zetasizer (PDF)

## Notes

The data that support the findings of this study are openly available in NTU research data repository DR-NTU (Data) at https://researchdata.ntu.edu.sg/privateurl.xhtml?token=b4783eec-4952-48dc-aae8-257bd2ec522f.
