# Supplementary material for: Lipid-Polymer Hybrid Nanoparticles Enhance the Potency of Ampicillin against Enterococcus faecalis in a Protozoa Infection Model
Source: ACS Infect Dis. 2021 Apr 19;7(6):1607–18. doi: 10.1021/acsinfecdis.0c00774 (PMC8383308; doi:10.1021/acsinfecdis.0c00774)
Supplement: Supplementary file 1 — id0c00774_si_001.pdf [file id0c00774_si_001.pdf]

Supporting Information for

Lipid-Polymer Hybrid Nanoparticles Enhance the Potency of Ampicillin against *Enterococcus faecalis* in a Protozoa Infection Model

Chuan Hao Tan*, Lai Jiang*, Wenrui Li*, Siew Herng Chan, Jong-Suep Baek, Noele Kai Jing Ng, Talgat Sailov, Sharad Kharel, Kelvin Kian Long Chong, Say Chye Joachim Loo #

* Contributed equally

#Correspondence to: joachimloo@ntu.edu.sg

This PDF file includes:

6 Pages; Figure S1 to S6

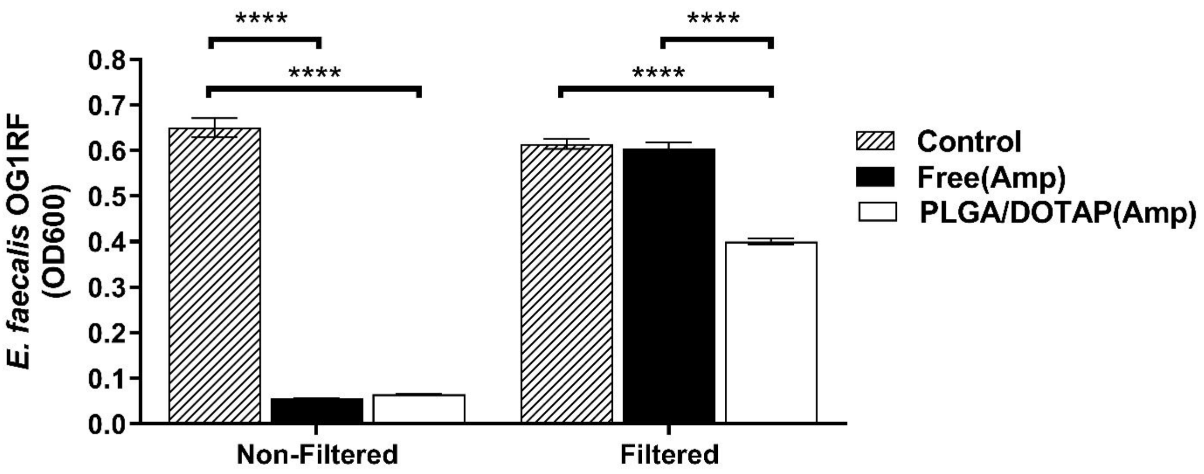

Figure S1. Residual Amp activity in the filter well, after filtration and washing, was assessed using the *E. faecalis* culture.

To assess whether the extracellular Amp in the filter wells was diffused out and diluted successfully, the residual Amp activity in the filter well, after medium exchange for 5 times, was determined using the *E. faecalis* culture. Nearly no bacteria grew in the non-filtered media containing either free Amp or Amp-LPNs, confirming their effectiveness against extracellular E. faecalis. No bactericidal effect was exhibited in the medium from the free Amp group after filtration and washing, indicating complete removal of extracellular free Amp. In the group pre-treated with Amp-LPNs, the medium after filtration and washing caused a reduction in the number of *E. faecalis* (indicated by the reduction in the OD reading), suggesting there was still Amp present in the 5extracellular environment. One-way ANOVA test with Tukey's multiple comparisons was performed. The statistical differences are indicated as follows: **** $P < 0.0001$.

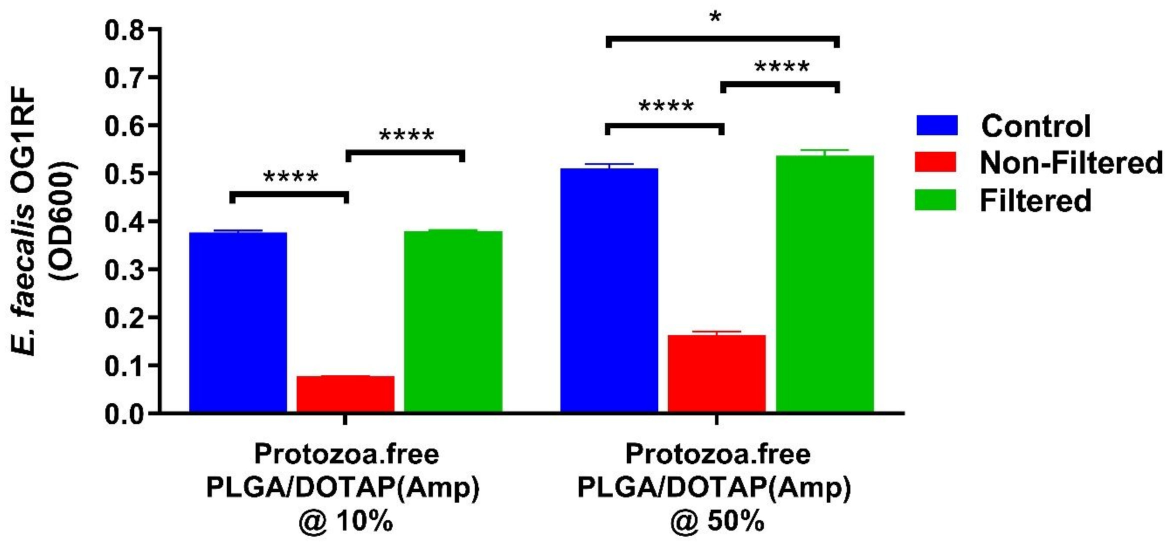

Figure S2. Residual Amp activity in a *T. pyriformis*-free control well, after filtration and washing, was assessed using the *E. faecalis* culture.

To determine whether Amp-LPNs presented in the extracellular environment in Fig. S1 was due to insufficient filtration/washing, the residual Amp activity after filtration and washing was assessed in a protozoa free control well. Filtered media even at 50% showed no bactericidal activity towards *E. faecalis*, confirming the effectiveness of our procedure in removing Amp in both free and encapsulated forms in the extracellular environment. One-way ANOVA test with Tukey's multiple comparisons was performed. The statistical differences are indicated as follows: * $P < 0.05$, **** $P < 0.0001$.

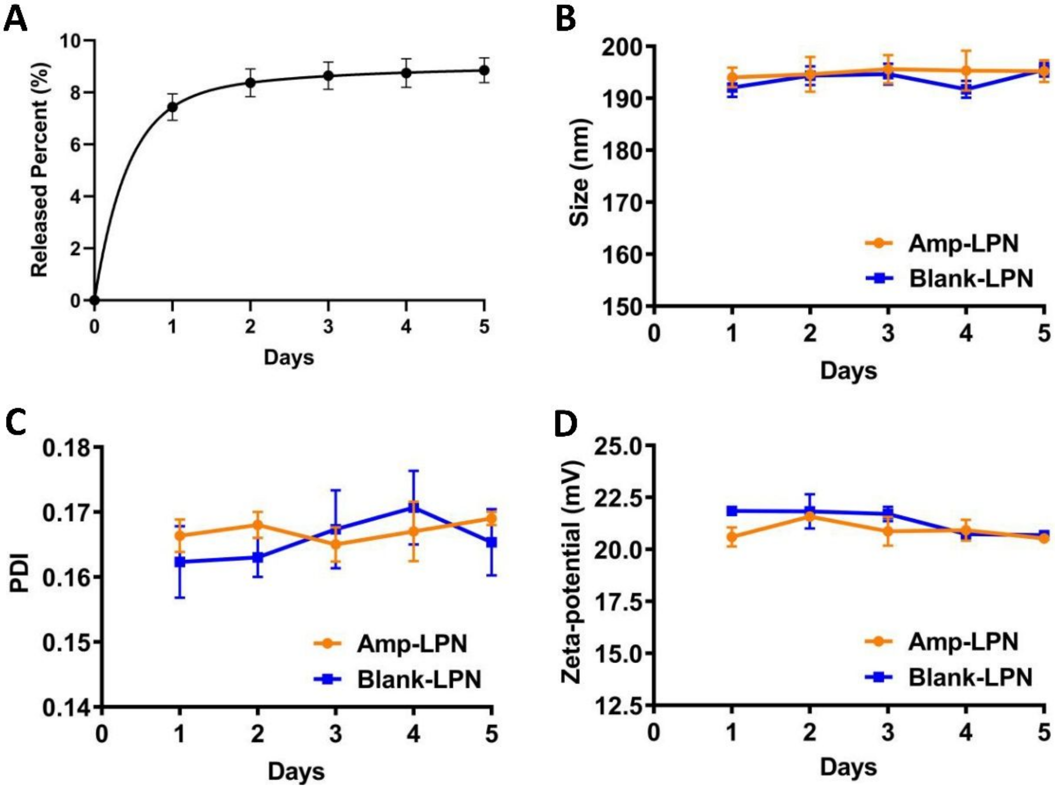

Figure S3. The characteristics of Amp-LPN and Blank-LPN. (A) the Release Profile of Amp-LPN in PBS (pH = 7). (B-D) the stability study under the same condition as release study, including (B) size (C) PDI (D) Zeta-potential. Mean ± SD, n = 3.

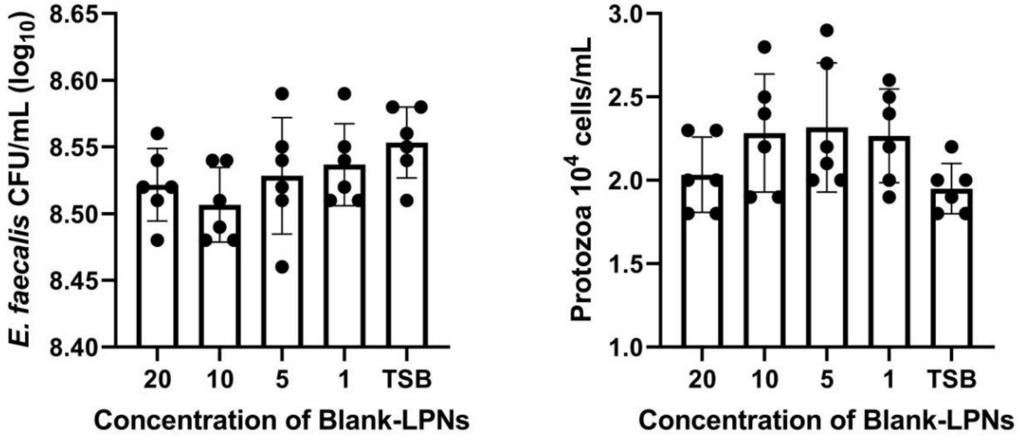

Figure S4. Toxicity test of blank LPNs (mg/mL) on *E. faecalis* (left) and protozoa (right). Mean ± SD, n = 6. No statistical significance was identified between groups receiving different concentrations of blank LPNs. Based on the drug loading (around 25 µg ampicillin/mg particles), the corresponding concentrations of Black-LPNs of maximum 250 µg/mL Amp in the report corresponds to 10 mg/mL Blank LPNs in the toxicity studies.

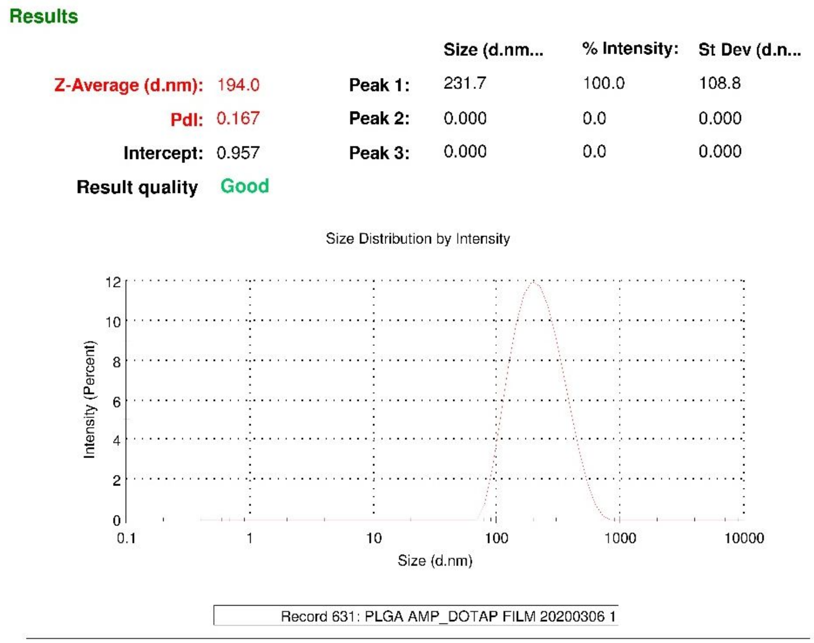

Figure S5. Raw data report of size distribution by Zetasizer.

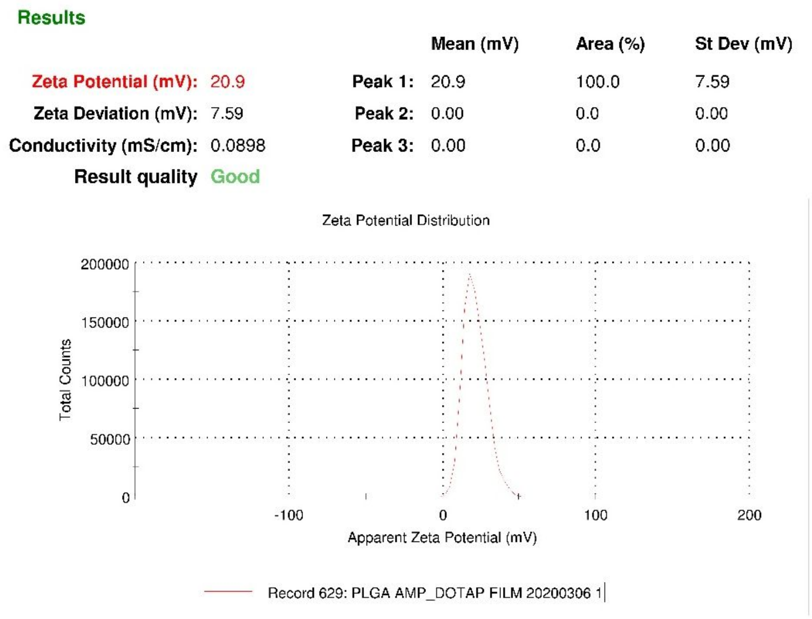

Figure S6. Raw data report of Zeta-potential distribution by Zetasizer.
